# Does erosion progress differently on teeth already presenting clinical signs of erosive tooth wear than on sound teeth? An in vitro pilot trial

**DOI:** 10.1186/s12903-016-0231-y

**Published:** 2016-07-07

**Authors:** Thiago Saads Carvalho, Tommy Baumann, Adrian Lussi

**Affiliations:** Department of Preventive, Restorative and Pediatric Dentistry, University of Bern, Freiburgstrasse 7, CH-3010 Bern, Switzerland

**Keywords:** Dental erosion, Assessment, Enamel, Optical reflectometer, Reflection analysis, Diagnostics, Calcium, Susceptibility

## Abstract

**Background:**

Erosive tooth wear (ETW) is clinically characterized by a loss of tooth surface, and different enamel depths may have different susceptibility to demineralization. Therefore, the aim of this in vitro pilot study was to assess if the progression of erosive demineralization is faster on teeth already presenting signs of ETW when compared to originally sound teeth.

**Methods:**

We selected 23 central incisors: 14 were clinically sound (Sound) and 9 presented clinical signs of early erosive tooth wear (ETW-teeth). The teeth were embedded in resin, leaving an uncovered window of native enamel (6.69 ± 2.30 mm^2^) on the incisal half of the labial surface. We measured enamel surface reflection intensity (SRI) initially and after each consecutive erosive challenge (1 % citric acid, total of 4, 8, 12, 16, 20 and 24 min). Calcium released to the citric acid was measured with an atomic absorption spectrometer.

**Results:**

We observed higher initial SRI values in ETW-teeth than in Sound teeth (*p* = 0.007). During in vitro erosive demineralization, we observed that erosion on originally Sound teeth progressed significantly slower (*p* = 0.033) than on ETW-teeth: SRI decreased by 75 % (from 100 to 25 %) on Sound teeth, and by 89 % (from 100 to 11 %) on ETW-teeth. Calcium release increased during erosion, but presented no significant differences (*p* = 0.643) between originally Sound (0.031 μmol/mm^2^) and ETW-teeth (0.032 μmol/mm^2^). There was satisfactory correlation between calcium release and rSRI values (*r*_*s*_ = −0.66).

**Conclusion:**

The optical reflectometer distinguished originally sound teeth from those with signs of ETW, and the results suggest that acid demineralization progresses differently on teeth already presenting clinical signs of ETW than on sound teeth.

**Electronic supplementary material:**

The online version of this article (doi:10.1186/s12903-016-0231-y) contains supplementary material, which is available to authorized users.

## Background

Erosive demineralization on enamel occurs both at the enamel/acid interface, as well as within a partly demineralised thin softened layer of its surface, in a process called near-surface demineralization [1]. Clinically, erosive tooth wear (ETW) is usually characterized by a loss of the natural morphology of the tooth surface [2]. Typical signs are: a shiny, silky-glazed, but sometimes dull, excessively smooth tooth surface, with the absence of perikymata [3, 4].

Enamel solubility to erosive demineralization can depend on different factors. Native enamel surfaces are usually less liable to acid dissolution than ground enamel surfaces [5, 6], and solubility has been shown to increase according to enamel depth [7]. In this case, the loss of the top layer of enamel (loss of perikymata) in teeth with initial signs of ETW may render them more liable to further erosive demineralization, but this still remains to be tested.

This in vitro pilot study aimed at assessing the progression of erosive demineralization on originally sound teeth in comparison to teeth presenting initial signs of ETW. The hypotheses tested were: initial SRI would differ between originally sound and originally eroded teeth; originally sound and originally eroded teeth would present different demineralization patterns after further in vitro erosion.

## Methods

### Enamel specimen selection and preparation

Twenty-three caries-free permanent human upper central incisors were used in the present experiment. The teeth were extracted by dental practitioners in Switzerland and they were kept in a 2 % Chloramine solution until the time of the experiment. Before the extraction, the patients were informed about the use of their teeth for research purposes and their oral consent was obtained. These teeth are from a pooled bio-bank, so the local ethics committee categorized the samples as “irreversibly anonymised”, and no previous approval was necessary. The present experiment was carried out in accordance with the approved guidelines and regulations of the local ethical committee (Kantonale Ethikkommission: KEK).

**Fig. 1:**
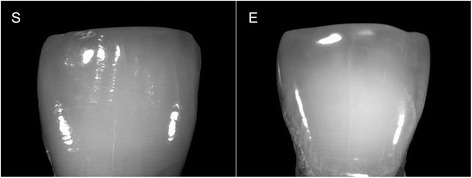
Photographs of an originally sound tooth (S) and a tooth that originally presented ETW (E)

The human teeth were cleaned and selected by a dentist (10 years experience), who examined the labial surfaces of the teeth under natural light. A total of 14 teeth without signs of ETW (sound), and 9 teeth with the typical signs of early ETW (silky, glossy, smooth enamel on the labial surface) were selected. Figure 1 illustrates the labial surfaces of teeth from both groups (S – originally sound teeth; E – teeth that already presented typical signs of early ETW). A section of the labial incisal half of the teeth was selected and embedded in acrylic resin (Paladur, Heraeus Kulzer GmbH, Hanau, Germany), leaving a window of native enamel. The surface area of this exposed enamel window was measured in all specimens. For that, images of the enamel surfaces were obtained using a light microscope (Leica, M420) at 12.5x magnification, connected to a camera (Leica, DFC495). Using the software program IM500, the contour of the exposed enamel window was traced and the surface area of each specimen was calculated (mean area of 6.69 ± 2.30 mm^2^, neglecting the natural curvature of the teeth).

### Experimental procedure and measurements

Initial enamel surface reflection intensity (SRI_0_) was measured on all specimens. The specimens were then exposed to a 4-min erosive challenge (7.5 ml of 1 % citric acid; pH = 3.6; 25 °C; shaking at 70 rpm), rinsed with deionized water (10 s), dried with oil-free air (5 s), and taken for another SRI measurement (SRI_1_). The citric acid used for erosion was reserved for later calcium analysis. A total of 6 consecutive erosive challenges were carried out (total of 24 min erosion), and after each erosion, SRI measurements (SRI_i_) and calcium analyses were made.

All SRI measurements were carried out using a prototype pen-size optical reflectometer [8]. For the measurements, the optical reflectometer was connected to a computer running a specific software that registers the point of highest reflection intensity, which is expressed as a SRI value. This SRI value is dimensionless, to which we attribute the unit *erf* (enamel reflection factor). In practical terms, higher SRI values represent smoother surfaces with greater reflection intensity [9, 10]. For statistical analyses, we considered the initial SRI values (SRI_0_), and the relative SRI (rSRI) after the erosive challenges. The latter was calculated using the formula rSRI_i_ = (SRI_i_/SRI_0_ * 100), where SRI_i_ is the value after the *i*^th^ erosive challenge (*i* = 1, 2, 3…6).

The calcium released into the citric acid was determined using an atomic absorption spectrometer with acetylene-air flame (AAnalyst 400, Perkin Elmer Analytical Instruments, USA). Standards of known calcium concentration were used to calibrate the instrument and obtain a working curve. Lanthanum nitrate was added to the citric acid and to the standards (final concentration 0.5 % w/v) to eliminate interference from phosphate ions. The amount of calcium released to the citric acid was measured and then normalized to the area of the enamel surface exposed to the acid (μmol/mm^2^).

To illustrate the effect of the acid on the enamel, scanning electron microscopy was carried out on two extra teeth (one sound and one with ETW), which underwent the same experimental procedure described previously. We took SEM stereo-pair images from the enamel surfaces. The stereo-pair images “a” and “b” are from the exact same surface: image “a” is before tilting, image “b” was taken after 15° tilting. Each stereo-pair were made from the same sound tooth (before and after the erosive challenges), or from the same tooth originally with ETW (before and after the erosive challenges). Magnification used was 300×, at 10 KV (JSM-6010PLUS/LV SEM, JEOL, Tokyo, Japan).

### Statistical analyses

The following null hypotheses were tested:

1. There is no difference in SRI_0_ between originally sound teeth and those presenting ETW;
2. There is no difference in total rSRI between originally sound teeth and those presenting ETW;
3. There is no difference in total calcium released between originally sound teeth and those presenting ETW.

Non-parametric ANOVA for repeated measures were also performed to analyse differences in rSRI and calcium release between the two groups throughout the experiment. Post-hoc Mann–Whitney U tests were used to verify the differences between groups in regards to SRI_0_ and rSRI (null hypotheses 1 and 2), as well as differences in total calcium release (null hypotheses 3). Within-group differences in rSRI between experimental times were analysed using Wilcoxon Sign Rank Test. In addition, we used Spearman rank correlation between the rSRI measurements and calcium results. Experimentwise probability for significance (α) was set at 0.05.

## Results

Teeth with signs of ETW presented significantly higher initial SRI values (SRI_0_) than originally sound teeth (*p* = 0.007). The median ± interquartile range for SRI_0_ for teeth with signs of ETW were 5.86 ± 2.84 erf and for originally sound teeth, 2.83 ± 2.82 erf. Since enamel specimens from teeth with typical signs of erosive wear presented significantly higher SRI_0_ values than originally sound teeth, null hypothesis 1 was rejected.

**Fig. 2:**
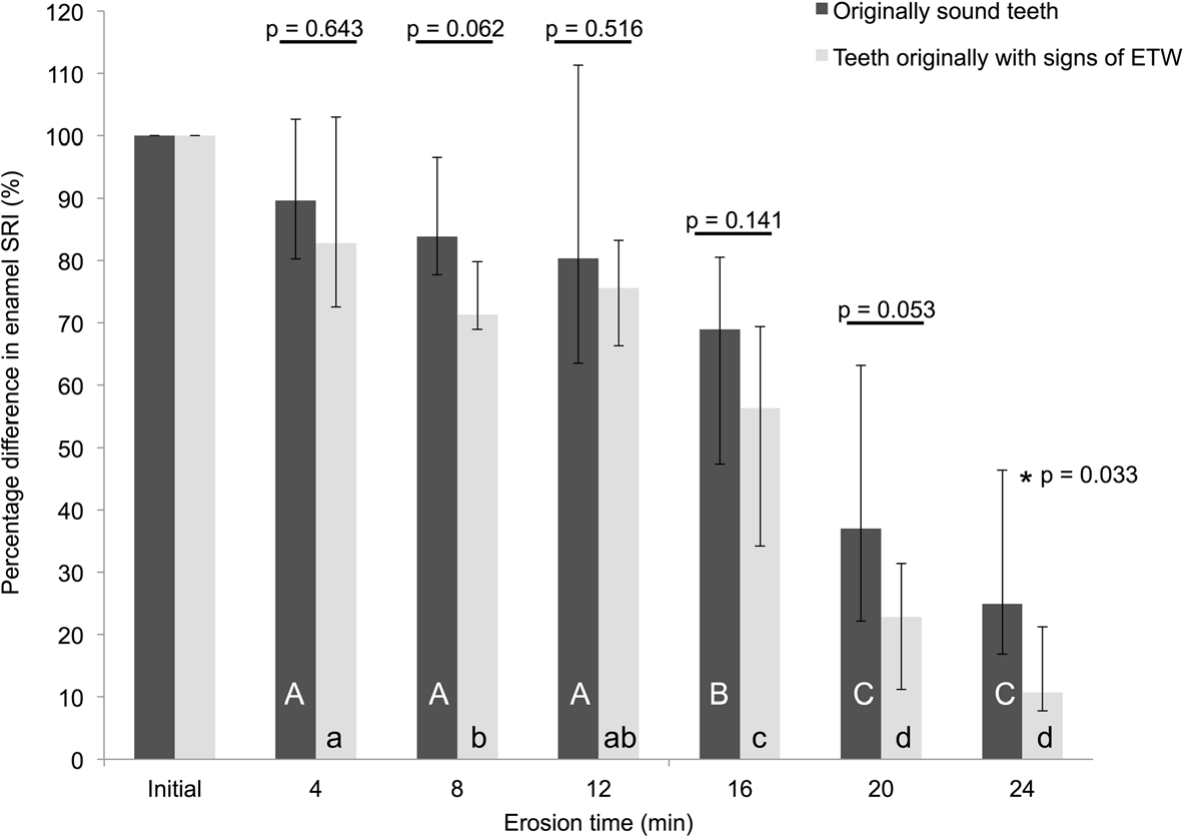
Median rSRI of originally sound teeth (black) and teeth that originally presented ETW (gray). Dispersion bars represent interquartile range. Different upper case letters represent differences between erosion times for originally Sound teeth, and different lower case letters represent differences between erosion times for teeth originally with ETW. P-values represent between-group differences (or lack thereof) at each time-point. For all analyses, no corrections for multiple testing were applied due to the explorative nature of the study

After further in vitro erosion, enamel SRI decreased to different extents in both groups. Erosive demineralization progressed faster on teeth that already presented clinical signs of ETW than on originally sound teeth (*p* = 0.028). Figure 2 shows the relative SRI (rSRI) throughout the experiment. After 24 min in vitro erosion, teeth with ETW presented significantly greater rSRI decrease than originally sound teeth (*p* = 0.033). We measured an rSRI decreased of 89 % (from 100 to 11 %) on teeth with ETW, and 75 % (from 100 to 25 %) on originally sound teeth. These significant differences between the groups led to the rejection of null hypothesis 2.

**Fig. 3:**
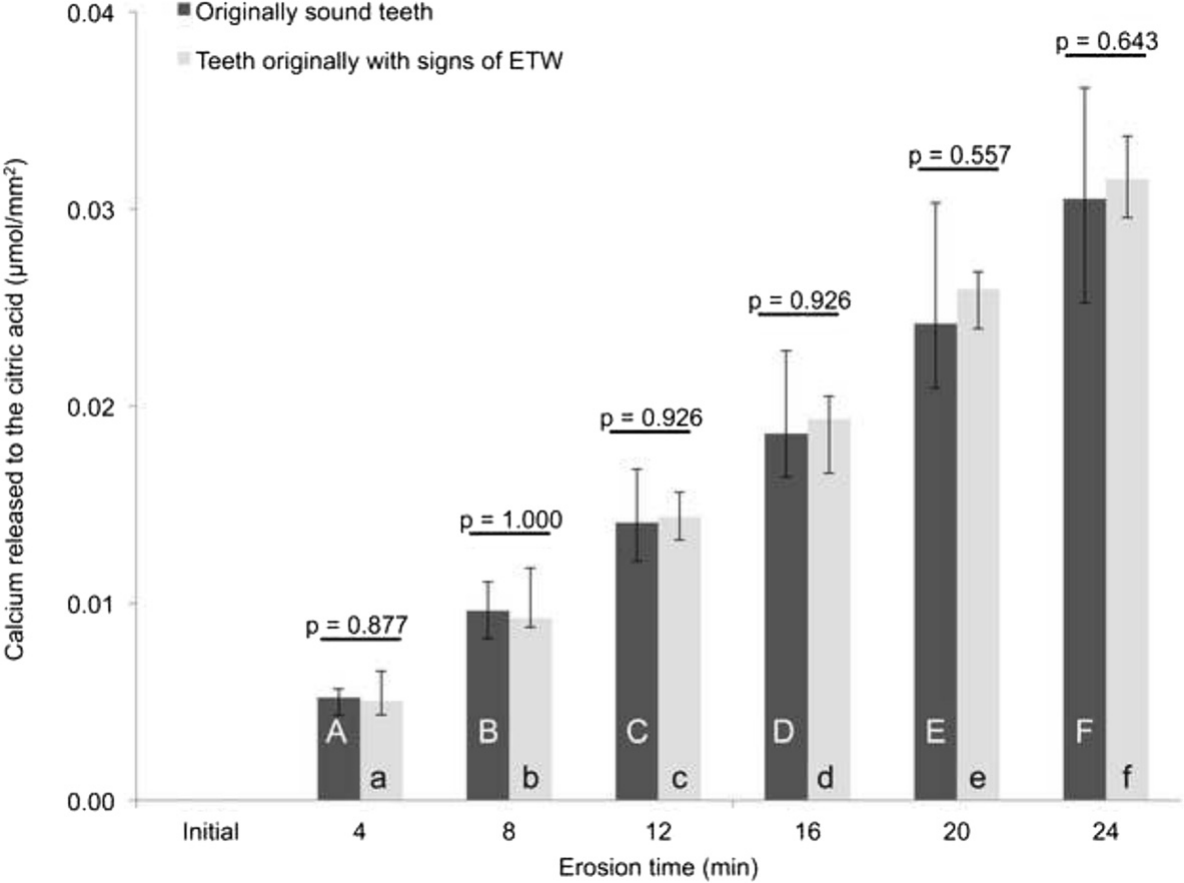
Median cumulative calcium released from originally sound teeth (black) and teeth that originally presented ETW (gray). Dispersion bars represent interquartile range. Different upper case letters represent differences between erosion times for originally Sound teeth, and different lower case letters represent differences between erosion times for teeth originally with ETW. For all analyses, no corrections for multiple testing were applied due to the explorative nature of the study

In relation to the calcium released during the erosive challenges (Fig. 3), no significant differences were observed between the originally sound teeth and teeth with ETW throughout the whole experiment (*p* = 0.724). After 24 min erosion, we also observed no differences in the total amount of calcium released (*p* = 0.643), where calcium values were 0.031 and 0.032 μmol/mm^2^, for originally sound teeth and teeth with signs of ETW, respectively. In this case, null hypothesis 3 was not rejected. However, there was satisfactory correlation between calcium release and rSRI values, with coefficient value of *r*_*s*_ = −0.66. Furthermore, considering each group separately, we observed lower correlation coefficient when only originally sound teeth were analysed (*r*_*s*_ = −0.61; *p* < 0.001), than when only teeth with signs of ETW were analysed (*r*_*s*_ = −0.83; *p* < 0.001).

**Fig. 4:**
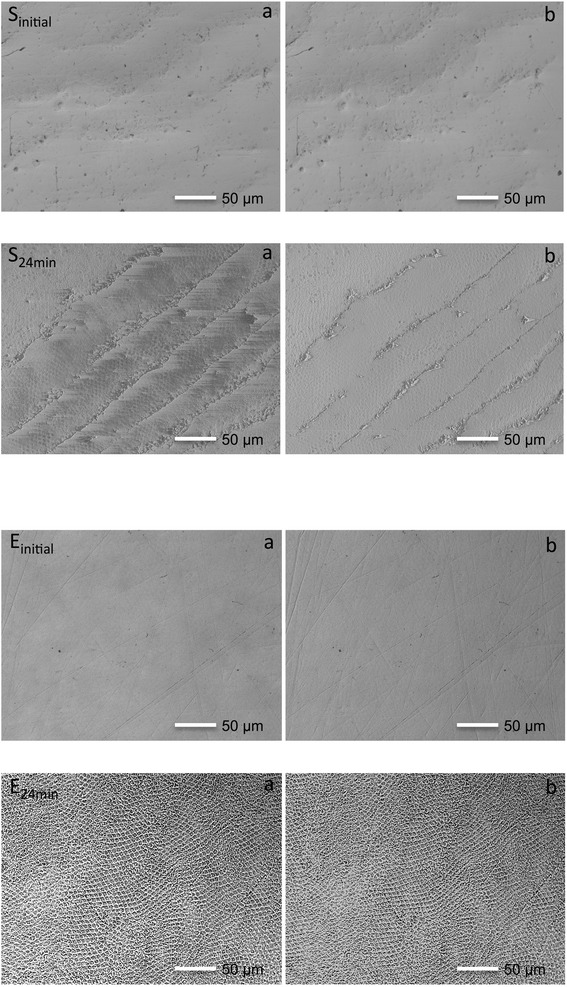
Scanning electron microscopy stereo-images of an originally sound tooth and a tooth originally with ETW. Figures a and b represent stereo-pair images taken from the exact same surface: image “a” was taken before tilting, and image “b” was taken after 15° tilting. Images were taken from the same enamel surface of an originally sound tooth (S) and a tooth that originally presented ETW (E), before (_initial_) and after (_24min_) in vitro erosion. The enamel specimens from originally sound teeth initially present clear undulations of the perikymata (S_initially_), which are absent on teeth that originally presented ETW (E_initially_). After in vitro erosion, teeth that originally presented ETW (E_24min_) exhibit the distinct characteristic of etched enamel

Figure 4 are stereo-pair images and serve as illustrations of the effect of the acid on the enamel surface relief and the micro-anatomy of originally sound (S) teeth and teeth with signs of ETW (E), at two time-points: before (_initial_) and after (_24min_) all acid challenges. Initially, the specimens of originally sound teeth presented the clear pattern of the perikymata relief, whereas on the teeth with ETW no perikymata were visible. After the acid challenges, the enamel from originally sound teeth still presented the contours of the perikymata with faint signs of enamel demineralization, whereas we clearly noticed the distinct honeycomb pattern of erosion on teeth with original signs of ETW.

## Discussion

**Fig. 5:**
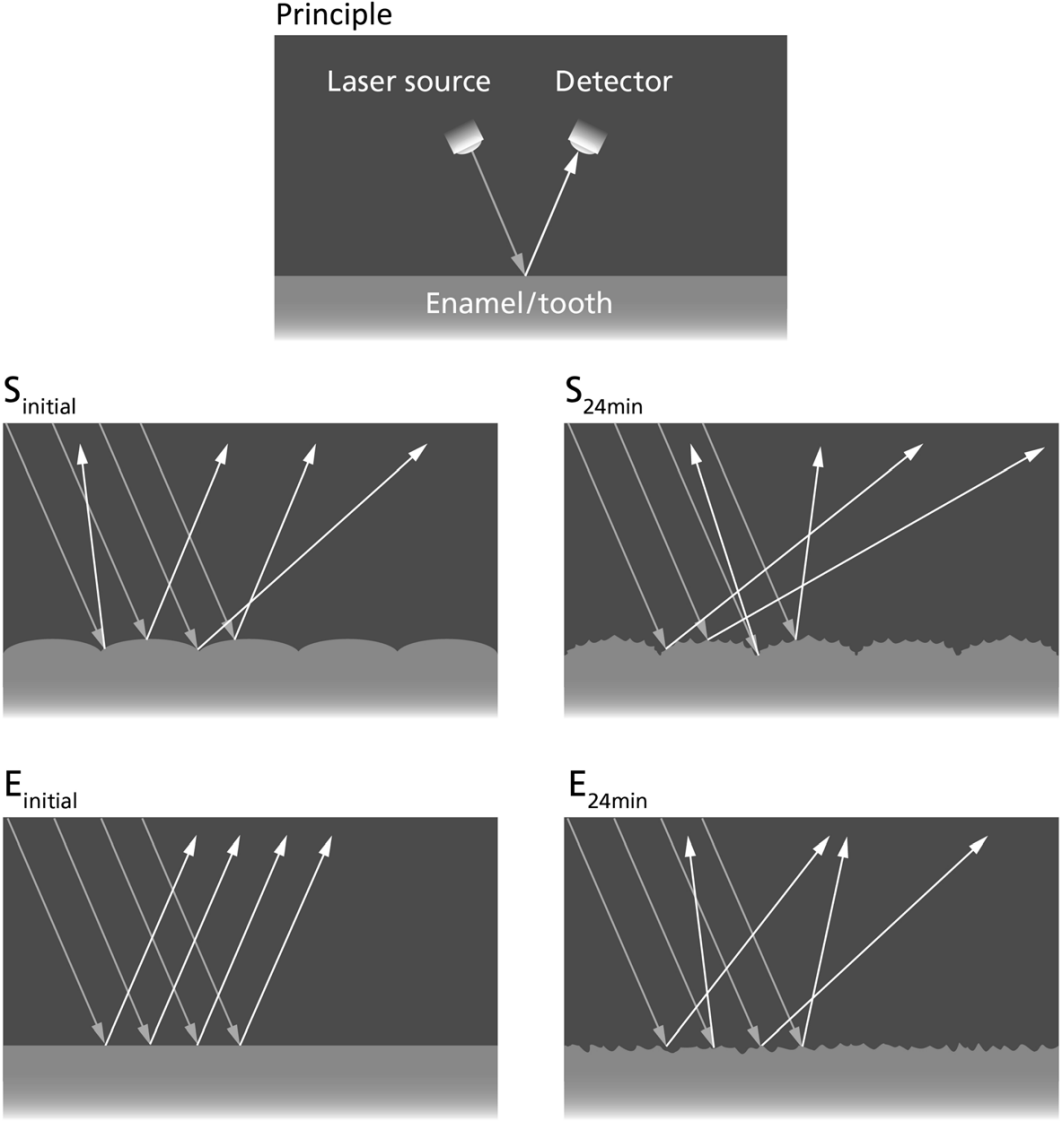
Schematic diagram of the optical principle of the reflectometer. Arrows show the incident and reflected light beams. The top diagram (Principle) represents the highest reflection intensity. The light shines at a ~23° angle onto the enamel/tooth surface and it reflects back also at a ~23° angle (specular reflection) for the highest surface reflection intensity (SRI). The other diagrams represent originally sound teeth (S) and teeth that originally presented ETW (E). Before acid challenges, S_initial_ has clear undulations of the perikymata that scatter some of the light, while E_initial_ has a flat smoother surface that reflects most of the light. So, in S_initial_ a smaller proportion of the reflected beams will be captured by the detector (lower SRI), while in E_initial_ a greater proportion of the light will be reflected back to the detector (higher SRI). After the erosive challenges (S_24min_ and E_24min_), the enamel surface is rougher, leading to more light scattering and, thus, lower SRI on both types of enamel (S and E)

The present study used the optical reflectometer to assess erosive demineralization. Previous studies had already shown that the optical reflectometer produces satisfactory results [8, 11, 12]. The principle of the optical reflectometer is based on the amount of light reflected back from the enamel surface (Fig. 5). The optical reflectometer shines a laser beam (wavelength: 635 nm) onto the enamel surface at an incidence angle of ~23°. This beam is reflected from the enamel surface also at a ~23° angle (specular reflection), and this reflected beam is then captured by the detector. In more practical terms, smoother enamel surfaces allow for a more regular reflection of the laser, hence they yield higher reflection intensity. Rougher enamel surfaces, however, tend to scatter the beam, and, hence, yield lower reflection intensity (Fig. 5) [12, 13].

In order to comprehend the results of this experiment, it is crucial to understand the histopathology of initial enamel erosion. First, when enamel comes in contact with the acid, the partial mineral dissolution causes an increase in surface roughness [14], leaving the enamel surface with decreased hardness (softening) and the typical honeycomb pattern reported by JH Meurman and RM Frank [15]. This “rougher” and “softer” enamel has a lower SRI [12] and is more vulnerable to mechanical forces, such as tooth brushing [16]. Abrasive forces partially remove the softened enamel layer and produce slightly smoother surfaces with increased SRI [10]. In the clinical situation, enamel is constantly submitted to these chemical and mechanical forces, so teeth with initial erosive tooth wear (ETW) are clinically characterized as having shiny, smooth surfaces, without perikymata [3, 4]. As reported previously [10, 12], enamel SRI is negatively correlated to surface roughness, hence smoother surfaces yield higher SRI values than rougher surfaces. Given that the teeth in the present study were selected according to their clinical aspects (Fig. 1), these surface topography characteristics explain the difference in initial SRI values between the groups (Fig. 5). The smoother aspects of enamel specimens from teeth that originally presented ETW yielded higher SRI_0_ values than the specimens from originally sound enamel [13].

After the erosive challenges, the SRI values continually decreased. This is in accordance with E Rakhmatullina, A Bossen, KK Bachofner, C Meier and A Lussi [8], who observed a continuous decrease in reflection intensity of native enamel with each subsequent incubation in orange juice. Interestingly, in our experiment, the enamel specimens from teeth that already presented ETW had a significantly greater rSRI decrease than the specimens from originally sound teeth after 24 min erosion. This is corroborated by the surface topography of the specimens, observed in Fig. 4. Although these SEM images (Fig. 4) serve only as illustrations, we see that, after the erosive challenges, there was a considerable change in surface topography on the teeth that originally presented ETW, in which we notice the typical honeycomb pattern of etched enamel. This pattern, however, is not as clearly detected on the originally sound enamel. Hence, we suggest that teeth with clinical signs of ETW are probably more prone to further acid demineralization than originally sound teeth.

The explanation for this difference could lie either on the different mineral composition of the surface enamel (perikymata), or on the genetic variation of the patients who donated the teeth. On the one hand, the specimens of teeth that originally presented ETW had already lost their perikymata, consequently, their outermost layer of enamel was already worn away. We have previously shown that different enamel depths have different solubility to erosion [17], so the dissolution of enamel can, therefore, be affected twofold. First, different enamel layers contain different mineral compositions [18], with outer layers having greater mineral density [19] and lower mineral solubility [7]. Secondly, originally sound teeth still have their perikymata, which might not dissolve as readily as prismatic enamel [15, 20], leading to no apparent alterations to the enamel surface after exposure to acid. Teeth that already present signs of ETW have lost their perikymata; their prism-heads are, therefore, more exposed to the acids, and are more liable to the effects of demineralization. Hence, we can observe the typical honeycomb structure after erosion. This suggests that once the prismatic enamel is reached, erosion progression will be faster. This was already observed by JH Meurman and RM Frank [15], who showed a distinct dissolution of prismatic enamel, but an irregular dissolution of the aprismatic surface. On the other hand, one should also consider the influence of genetic components on enamel solubility. One example is the study by JB Sovik, AR Vieira, AB Tveit and A Mulic [21] that recently observed an overrepresentation of the G allele of an enamelin marker in the saliva from patients presenting signs of erosive tooth wear. The authors suggested that genetic variations contribute to alterations in the enamel mineral structure, and, hence, it can lead to a greater susceptibility to erosive demineralization [21].

Taking the calcium results into consideration, we detected no differences between the two groups. However, the distinctions observed in the rSRI values and in the SEM images are still plausible. The citric acid used for the erosive challenges in the present experiment was completely unsaturated with respect to all fractions of the enamel mineral. This led to a maximum rate of dissolution for all specimens at all erosion times [22]. This means that the citric acid reacted to its maximal potential with all enamel surfaces, thus removing similar amounts of mineral from both originally sound teeth and teeth that originally presented ETW. Also, the presence of impurities in the crystal lattice of deeper enamel layers could affect these results. In deeper enamel layers, there is greater concentration of carbonated hydroxyapatite crystals [23], which have a more distorted structure and can accommodate more magnesium [24]. Magnesium, in turn, appears as a substituent for calcium; hence less calcium would be detected in deeper layers. It is suggested that the analysis of phosphate release in further experiments could clarify this effect, and possibly allow observations of subtler differences between the two surfaces. Still, notwithstanding the lack of difference in the calcium release values, the physical changes occurring during erosion were distinct on the different enamel surfaces. It is important to bear in mind that chemical analyses of calcium, albeit precise to measure erosive demineralization, does not provide any information on the physical and morphological changes of the enamel surfaces [25]. So all these changes were actually measured by the optical reflectometer, and can be observed with the SEM.

Despite the fact that we found no differences in calcium released from originally sound and teeth that originally presented ETW, we have observed a significant correlation between the calcium and rSRI results (*r*_*s*_ = −0.66; *p* < 0.001). Additionally, we also observed different correlation coefficient values when we analysed results only from sound teeth (*r*_*s*_ = −0.61; *p* < 0.001) or from teeth that already presented signs of ETW (*r*_*s*_ = −0.83; *p* < 0.001). One reason for this could be the presence of perikymata in originally sound teeth that causes greater variation in SRI measurements, and hence lower correlation values with the calcium results. On the other hand, the smoother aspects of teeth that already presented signs of ETW lead to a more regular (higher) reflectivity, which, in turn, causes less variation in SRI measurements, and hence higher correlation values with the calcium results. Previous studies with highly polished (smoother) enamel surfaces also presented satisfactory correlation with calcium release [11, 12]. Furthermore, although our general correlation coefficient value (*r*_*s*_ = −0.66) is lower than that reported by E Rakhmatullina, A Bossen, C Höschele, X Wang, B Beyeler, C Meier and A Lussi [12], it is in a similar range as that reported by SC Brevik, A Lussi and E Rakhmatullina [11]. In any case, calcium release and the optical reflectometer assess different experimental parameters. The former focuses on the analysis of minerals released from enamel during erosion, while the latter is more closely related to the surface texture of enamel. Recently, AT Hara, SV Livengood, F Lippert, GJ Eckert and PS Ungar [26] showed that some devices are able to differentiate the complex surface textures of erosion and erosion-abrasion lesions. So, further studies are still necessary to verify how the reflectometer differentiates different kinds of lesions (erosion, abrasion or erosion-abrasion) on both enamel and dentine.

## Conclusions

For each null hypotheses tested, we conclude the following:

1. We rejected null hypothesis 1, showing that there is difference in SRI_0_ between originally sound teeth and those presenting ETW;
2. We rejected null hypothesis 2, where we observed a difference in total rSRI between originally sound teeth and those presenting ETW;
3. We accepted null hypothesis 3, that there is no difference in total calcium released between originally sound teeth and those presenting ETW.

We, therefore, conclude that initial SRI significantly differs between originally sound and originally eroded teeth, and that the teeth that already had signs of ETW present different demineralization patterns than originally sound teeth after further in vitro erosion.

## Abbreviations

*erf*, enamel reflection factor; ETW, erosive tooth wear; rSRI, relative surface reflection intensity; SEM, scanning electron microscopy; SRI, surface reflection intensity; SRI_0_, initial (baseline) surface reflection intensity

## Additional file

Additional file 1: Data. (XLSX 54 kb)
